# Supplementary material for: Altered microRNA Transcriptome in Cultured Human Liver Cells upon Infection with Ebola Virus
Source: Int J Mol Sci. 2021 Apr 6;22(7):3792. doi: 10.3390/ijms22073792 (PMC8038836; doi:10.3390/ijms22073792)
Supplement: Supplementary file 1 [file ijms-22-03792-s001.zip › Supplementary_File/C_ GO_Analysis_Results/16-30nt_go_Mayinga-24h-Huh7_vs_Control-24h-Huh7_up.mature_mirna_targets/BP_EnrichmentScore.pdf]

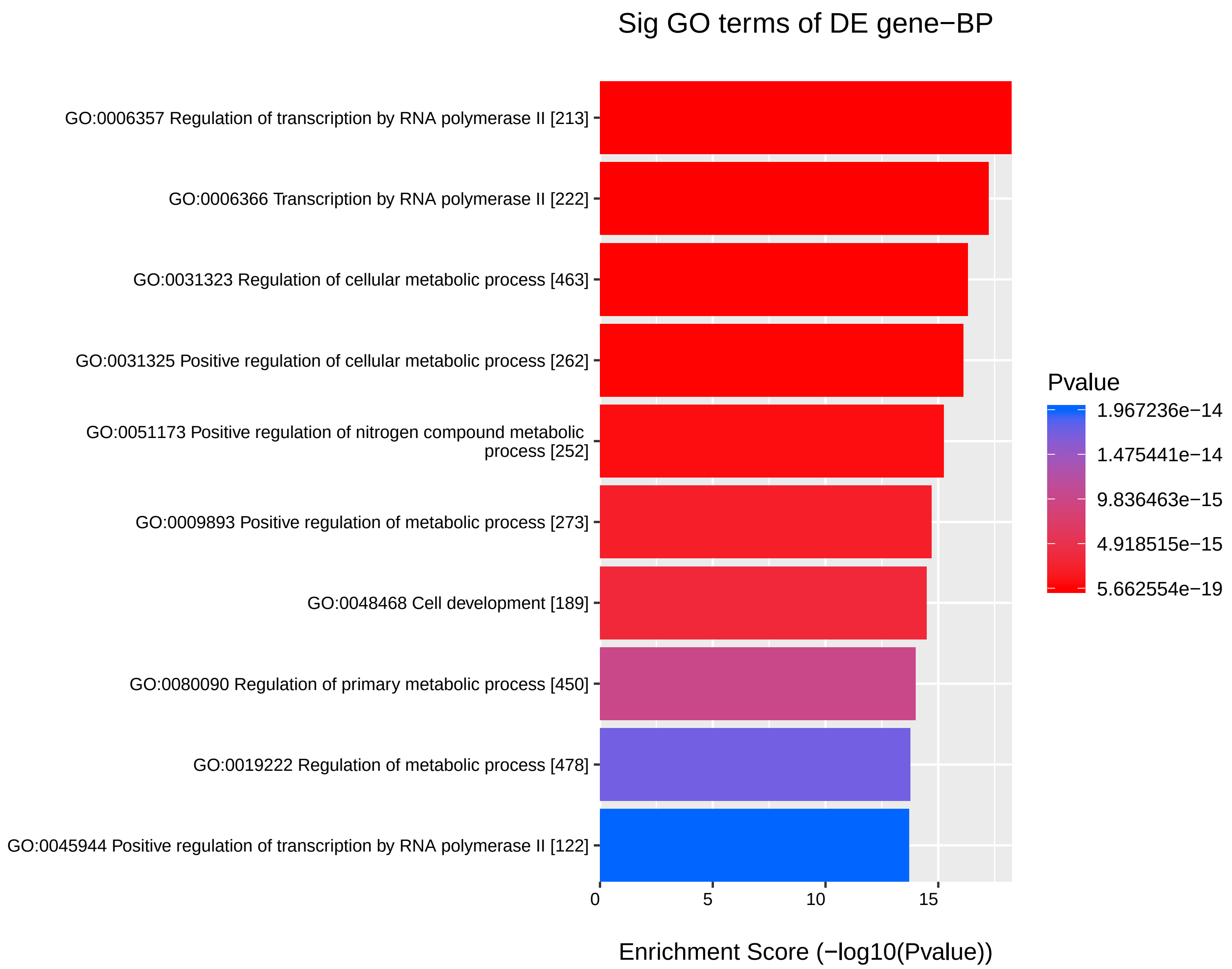

Sig GO terms of DE gene−BP
GO:0006357 Regulation of transcription by RNA polymerase II [213]
GO:0006366 Transcription by RNA polymerase II [222]
GO:0031323 Regulation of cellular metabolic process [463]
GO:0031325 Positive regulation of cellular metabolic process [262]
GO:0051173 Positive regulation of nitrogen compound metabolic process [252]
GO:0009893 Positive regulation of metabolic process [273]
GO:0048468 Cell development [189]
GO:0080090 Regulation of primary metabolic process [450]
GO:0019222 Regulation of metabolic process [478]
GO:0045944 Positive regulation of transcription by RNA polymerase II [122]
0
5
10
15
Enrichment Score (−log10(Pvalue))
Pvalue
1.967236e−14
1.475441e−14
9.836463e−15
4.918515e−15
5.662554e−19
